# Secure Cooperative Spectrum Sensing for the Cognitive Radio Network Using Nonuniform Reliability

**DOI:** 10.1155/2014/101809

**Published:** 2014-09-11

**Authors:** Muhammad Usman, Insoo Koo

**Affiliations:** Department of Electrical, Electronics and Computer Engineering, University of Ulsan, 93 Daehak-ro, Nam-gu, Ulsan 680-749, Republic of Korea

## Abstract

Both reliable detection of the primary signal in a noisy and fading environment and nullifying the effect of unauthorized users are important tasks in cognitive radio networks. To address these issues, we consider a cooperative spectrum sensing approach where each user is assigned nonuniform reliability based on the sensing performance. Users with poor channel or faulty sensor are assigned low reliability. The nonuniform reliabilities serve as identification tags and are used to isolate users with malicious behavior. We consider a link layer attack similar to the Byzantine attack, which falsifies the spectrum sensing data. Three different strategies are presented in this paper to ignore unreliable and malicious users in the network. Considering only reliable users for global decision improves sensing time and decreases collisions in the control channel. The fusion center uses the degree of reliability as a weighting factor to determine the global decision in scheme I. Schemes II and III consider the unreliability of users, which makes the computations even simpler. The proposed schemes reduce the number of sensing reports and increase the inference accuracy. The advantages of our proposed schemes over conventional cooperative spectrum sensing and the Chair-Varshney optimum rule are demonstrated through simulations.

## 1. Introduction

The increasing demand for wireless services has driven the need for intelligent allocation and efficient use of the wireless spectrum. Conventional spectrum allocation results in spatiotemporal underutilization and scarcity of the spectrum. According to the Federal Communications Commission (FCC), the spatial and temporal variations in the utilization of the assigned spectrum range from 15% to 85% [1, 2].

Cognitive radio (CR) technology has been proposed to combat the spectrum shortage problem by allowing the opportunistic use of the wireless spectrum, which is primarily allocated to primary (licensed) users (PU), by secondary (unlicensed) users (SUs) under a given level of interference to the PU [3, 4]. Such a scheme requires the SU to detect the PU signal accurately and quickly [5]. Some of the various techniques used for spectrum sensing are energy detection, cyclostationary detection, matched filter detection, wavelet detection, and covariance detection. Energy detection is the method of choice due to its computational simplicity and ease of implementation, as well as its minimal requirement of prior knowledge of the primary signal. However, sensing performance of a single SU is greatly affected by the destructive channel effects such as shadowing and fading, thereby hindering the ability of the SU to distinguish between a deep fade and white space. Cooperative spectrum sensing (CSS) is used to overcome the channel effects and exploit location diversity to detect even a weak primary signal [6].

The presence of a malicious user (MU) deteriorates the detection performance of cooperative spectrum sensing. An MU is an unwelcome and unauthorized user who impersonates a legal user and propagates false information about the status of the primary signal. Generally known types of MUs include always busy (AB), always free (AF), always opposite (AO), and an MU that transmits high signal with probability *α* and low signal with probability 1 − *α*, and we name it *α*MU. In AB and AF types, an MU always generates either a high (*H*_1_) or a low (*H*_0_) signal, respectively, regardless of the actual status of the primary signal. In the case of AO, an MU always generates a signal about the status of the PU that is opposite of its local observation. The AO MU is considered to be the most dangerous type, especially, when the decision is taken opposite to the real status of PU (if global decision or real status of the PU is known).

Cooperative sensing can improve the detection and false alarm probabilities [7], however, a high number of cooperative users, where majority of users have low SNR, may not produce optimal performance [8] and may have a negative impact on the complexity of the network, sensing time (latency), control channel bandwidth, collision in the control channel, and energy consumption. The number of SUs can be controlled by assigning reliability to them according to their sensing reports. Such reliability is based on correlation with the global decision. Users may send a deviant result due to either channel effects or malfunctioning of the sensors. The consistently deviant users are excluded from participation in the global decision, which leaves fewer but only reliable users in the network. Three different schemes are proposed in this paper to identify and remove consistently unreliable users and MUs which results in a less complex network consisting of fewer and more reliable nodes, which in turn reduces the computational burden on the fusion center (FC) and decreases the latency and overall energy consumption of the network.

Cooperative spectrum sensing increases the sensing performance of a CR network by using the location diversity of SUs [7]. However, presence of even few MUs severely degrades the performance of CSS. In [8], the authors have shown that a certain number of users (not all the users) with highest SNR achieve optimal sensing performance. However, the authors do not consider malicious behavior of the SUs and the decision of fusion center is solely based on high SNR users even if they report false data. To nullify the effect of MUs, reputation-based CSS with assistance from trusted nodes has been considered [9]. In [10], a statistical model of the PU was used in a soft reputation-based secure CSS scheme. Such an approach utilizes assistance from trusted nodes in the network. The assumption of trusted nodes is not practical due to the unavailability of such nodes in most cases. Furthermore, the significance of cooperative spectrum sensing is reduced if trusted nodes are the primary source for a result. In [11], an extended sequential CSS scheme was used in which SUs were polled to send their sensing result according to their reputation order. Uniform and fixed reputation degrees were employed for CUs in [12], while uniform reputation with no MU was used in [13]. In all of the above-cited studies, uniform reliability was assigned to users regardless of whether they produce good, normal, or bad results. Furthermore, only two types of MUs (AB and AF) were considered. None of the studies has addressed *α*-based MU and AO, the most dangerous types of MU.

In our previous work [14], the decision of disengagement of an SU and an MU, of types AO and AB, is taken by the FC based on reliability of the SU. In this paper, we extend our work by proposing three different schemes to deal with unreliable and malicious users. We also mitigate the effect of the MU that transmits high and low PU status based on probabilistic parameter *α*. In the first two schemes, an identification tag (IT) is used to restrict MUs, while reliabilities and unreliabilities are used to isolate unreliable users. The IT represents the reliability value of each user. It is calculated on the basis of correlation between the result of each user and FC and is communicated to the SUs in encrypted form. Unauthorized or malicious user would be unable to decrypt the IT. In the third scheme, the detection performance depends on honesty of the users. Dishonest and MUs severely degrade the performance of the network. Our proposed schemes are advantageous due to their computational simplicity, which makes them more practical and easy to implement. With a lower number of users and an avoidance of complex algorithms, the proposed approaches produce results that are comparable to (in terms of detection performance) and better than (in terms of the number of users) those obtained with the Chair-Varshney scheme and better (in all aspects for certain types of MUs) than those attained with the conventional CSS technique.

The remainder of this paper is organized as follows. The system model is described in Section 2, and our proposed schemes are presented in Section 3. Simulation results and discussion are given in Section 4. Conclusion is presented in Section 5.

## 2. System Model

**Figure 1:**
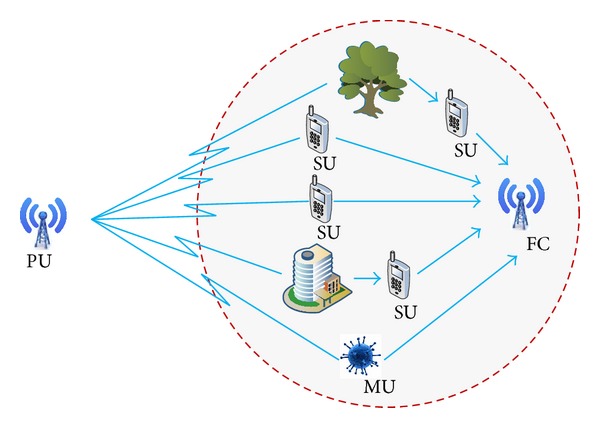
Cooperative users in a CR network.

We consider a network consisting of one PU and *N* SUs with *M*  (*M* ≤ *N*) reliable users and *L* malicious users such that 0 ≤ *L* ≪ *M*, shown in Figure 1. The remaining *N* − *M* − *L* users are unreliable users. Initially *M* is equal to *N* (if there is no MU); however, with the training of the CR network, *M* gets smaller than *N* due to disappearance of the unreliable users but remains above a minimum threshold, *N*_min⁡_. The maximum number of MUs is *L*_max⁡_. The number of reliable users (users with a good channel) is assumed to be more than the number of unreliable users (users with a poor channel) and MUs. Each MU may adopt one of the malicious modes described earlier. We consider an *m*-bit error-free common control channel between the SU and FC.

Detection of the primary signal is a binary hypothesis testing problem. The signal received by the *i*th SU is given as

(1) $$\begin{array}{c} H_{0}\text{:}\;x_{i}\left(n\right)=u\left(n\right),\;i=1,2,\ldots ,N,\\ H_{1}\text{:}\;x_{i}\left(n\right)=h_{i}\left(n\right)s\left(n\right)+u\left(n\right),\;n=1,2,\ldots ,S, \end{array}$$

where *H*_0_ and *H*_1_ correspond to the hypotheses that the PU signal is absent and present, respectively, *s*(*n*) represents the primary signal received at the SU, *h*_*i*_(*n*) is the amplitude gain of the channel, *u*(*n*) is the additive white Gaussian noise (AWGN) with zero mean and *σ*_*u*_^2^ variance, *N* is the number of SUs, and *S* is the number of samples. We assume that *s*(*n*) and *u*(*n*) are completely independent. Without loss of generality, the variance of noise is assumed to be the same at every sensor.

Each SU uses *S* samples in the sensing interval to perform spectrum sensing using the energy detection technique [15]. The local observation of the *i*th user is given by

(2) $$\begin{array}{c} y_{i}=\overset{S}{\underset{n\;=\;1}{\sum }}{\left|x_{i}(n)\right|}^{2}, \end{array}$$

where *S* is the number of samples and is equal to 2*TW*, and *T* and *W* are the sensing time and bandwidth, respectively. When *S* is relatively large (e.g., *S* > 200), *Y*_*i*_ can be well approximated as a Gaussian random variable under both hypotheses *H*_0_ and *H*_1_ with means *μ*_0_, *μ*_1_ and variances *σ*_0_^2^, *σ*_1_^2^, respectively, as follows [16]:

(3) $$\begin{array}{c} H_{0}\text{:}\;\mu_{0}=S\sigma_{u}^{2},\;\;\sigma_{0}^{2}=2S\sigma_{u}^{4},\\ H_{1}\text{:}\;\mu_{1}=S\left(\gamma_{i}+1\right)\sigma_{u}^{2},\;\;\sigma_{1}^{2}=2S\left(2\gamma_{i}+1\right)\sigma_{u}^{4}, \end{array}$$

where *γ*_*i*_ is the signal-to-noise ratio (SNR) of the primary signal at the* i*th SU. In each time slot, the FC broadcasts a request to all SUs to perform local sensing. After the sensing period, each SU reports its observation to the FC in the reporting period. The FC combines the received local observations and makes a global decision. We assume that the global decision taken by the FC is correct all of the time. The FC also computes the reliability of each user based on the compliance of an SU's local observation with the global result. Finally, the global decision along with respective reliability in the encrypted form as identification tag is communicated to each user.

Authentication is an integral component of the security protocols [17–19]. A three-stage security protocol consisting of prevention, detection, and cure is proposed in [17]. The prevention stage includes authentication and authorization; the participating users and their data will be authenticated in the authentication stage while recognition of the users is performed in the authorization stage. In [18], the authors proposed remote based smart card authentication scheme where an additional security stage called registration is introduced, in which details of users along with specific details given by the server are stored. In [19], a lightweight authentication scheme is used to guarantee security and privacy in global mobility networks. In [20], basic and extended features are used to detect malicious activity by applying adaptive support vector machines. In [21], a cryptographic technique like blind signature and electronic coin is used to achieve mobility, reliability, anonymity, and flexibility in a mobile wireless network. In this paper, we use an encrypted identification tag for the authentication of users and reliability test for the detection of unreliable and malicious users. The identification tag is assigned to users based on their reported observations.

## 3. Secure Reliability-Based CSS 

In conventional CSS, each SU performs local sensing and forwards either its quantized local observation *y*_*i*_ ((2) in the case of a soft decision) or local decision *H*_1_ or *H*_0_ ((4) in the case of a hard decision) to the FC through a dedicated control channel. Here,

(4) $$\begin{array}{c} y_{i}\frac{\overset{H_{1}}{>}}{\underset{H_{0}}{<}}\lambda_{i},\;i=1,2,\ldots ,N, \end{array}$$

where *λ*_*i*_ is the local energy threshold at the *i*th SU. The detection performance of the CR network is measured by the probability of detection *P*_*d*_ which is a measure of the interference to the PU and the probability of false alarm *P*_*F*_ which sets the upper bound on spectrum utilization. A higher value of *P*_*d*_ will protect the quality of service (QoS) of the PU, and a lower value of *P*_*f*_ will result in higher spectrum utilization. The detection and false alarm probabilities of the *i*th user are given, respectively, as

(5) $$\begin{array}{c} P_{d,i}=P\left(y_{i}>\lambda_{i}\;|\;H_{1}\right)=Q\left(\frac{\lambda_{i}-S\left(\gamma_{i}+1\right)\sigma_{u}^{2}}{\sigma_{u}^{2}\sqrt{2S\left(2\gamma_{i}+1\right)}}\right),\\ P_{f,i}=P\left(y_{i}>\lambda_{i}\;|\;H_{0}\right)=Q\left(\frac{\lambda_{i}-S\sigma_{u}^{2}}{\sigma_{u}^{2}\sqrt{2S}}\right), \end{array}$$

where *Q*(·) is a monotonically decreasing function defined as $Q(x)=(1/\sqrt{2\pi })\int_{x}^{\infty }\exp\left(-t^{2}/2\right)dt$. Sensing results from several SUs are combined at the FC as weighted sum and given as

(6) $$\begin{array}{c} Z=\overset{M}{\underset{i\;=\;1}{\sum }}w_{k-1}^{i}\times y_{i}, \end{array}$$

where *w*_*k*−1_^*i*^ is the weighting coefficient or reliability of the *i*th SU in the previous slot which is computed in Section 3.1.1; it is used to highlight or suppress the result of a certain SU based on detection performance. Finally, the status of the primary signal is determined as

(7) $$\begin{array}{c} Z<\lambda ,\;H_{0},\\ Z\geq \lambda ,\;H_{1}, \end{array}$$

where *λ* is the global threshold. The global detection and false alarm probabilities are expressed as

(8) $$\begin{array}{c} P_{D}=P\left(Z>\lambda \;|\;H_{1}\right)=Q\left(\frac{\lambda -S\sum_{i\;=\;1}^{M}w_{i}\left(\gamma_{i}+1\right)\sigma_{u}^{2}}{\sqrt{2S\sum_{i\;=\;1}^{M}w_{i}^{2}\left(2\gamma_{i}+1\right)\sigma_{u}^{4}}}\right),\\ P_{F}=P\left(Z>\lambda \;|\;H_{0}\right)=Q\left(\frac{\lambda -S\sum_{i\;=\;1}^{M}w_{i}\sigma_{u}^{2}}{\sqrt{2S\sum_{i\;=\;1}^{M}w_{i}^{2}\sigma_{u}^{4}}}\right), \end{array}$$

respectively.

### 3.1. Our Proposed Schemes

**Figure 2:**
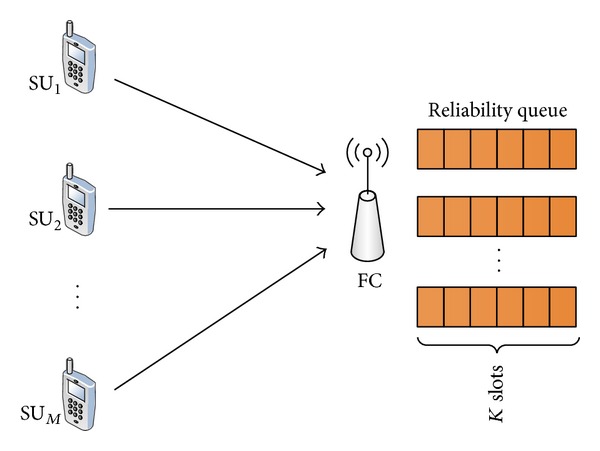
Reliability queue at the fusion center.

It is assumed that FC maintains *M* queues which are collectively called the reliability queue and is represented by *Q*, as shown in Figure 2. The size of each queue is *K* which denotes the previous history of reliability maintained for each of the reliable SUs. The value of *K* reflects a trade-off between the sensing accuracy and speed. FC receives sensing results from all SUs with equal initial reliability which is updated based on the distance between the local sensing result and the global decision in scheme I. The SU that produces more congruent result with respect to the global decision is assigned a higher reliability and vice versa. In contrast, schemes II and III use unreliability, instead of reliability, to evaluate an SU for participation in the global decision. An MU is detected and isolated from the network in schemes I and II because FC takes the decision by a two-tier checking process. However, the decision of disengagement from the network is taken by SUs (not FC) themselves and thus, an MU cannot be detected in scheme III. In this case, the fusion center relies upon the rectitude of the SUs.

#### 3.1.1. Proposed Scheme I

Rather than using complex calculations to compute the reliability of SUs, a simple method is proposed in this study. Each SU performs local sensing in the sensing period and forwards its observation to the FC in the reporting period. FC accepts the receiving data from the SUs with equal initial reliability and takes a global decision using data fusion (soft decision) technique. The initial reliability (weight) can be assigned to each SU as discussed in [10] but for simplicity, in this work, we assign equal initial reliability to the SUs that makes the initial weighting coefficient equal in (6) for each SU's report. The channel condition between PU and SU is then quantified into reliability which is measured on the basis of how much the SU supports or deviates from the global result. Based on the reliabilities in the previous slot and reports from the users in the current slot, FC takes the global decision. In (6) weights of all the SUs are taken into account for the global decision. However, to calculate/update weight of the *i*th SU, local observations of all SUs except the *i*th SU should be considered in order to minimize bias of the *i*th SU in weights assignment [22]. In [22, 23] the authors update the weight coefficients using the Chair-Varshney technique. However, in practical scenarios the detection and false alarm probabilities are not known a priori. Further, they do not handle the malicious users. In this work, we propose the update of weights based on the reported observations of the SUs. The global decision, excluding the *i*th SU, can be computed as below:

(9) $$\begin{array}{c} Z_{i}=\overset{M}{\underset{j\;=\;1}{\sum }}w_{k-1}^{j}y_{j}-w_{k-1}^{i}y_{i}=\overset{M}{\underset{j\;=\;1,j\;\neq \;i}{\sum }}w_{k-1}^{j}y_{j}. \end{array}$$

The set of all energies reported by the SUs is represented by *Y* as

(10) $$\begin{array}{c} Y=\left\{y_{1},y_{2},\ldots ,y_{M}\right\}. \end{array}$$

To update weight of the *i*th SU, *M* − 1 users are considered by excluding the *i*th SU as follows:

(11) $$\begin{array}{c} Y_{i}^{o}\subset Y=\left\{y_{l}:l=1,2,\ldots ,M,l\neq i\right\}, \end{array}$$

where *Y*_*i*_^*o*^ is the set of energies of all SUs except the *i*th SU. *Y*_*i*_^*o*^ is sorted into the ordered set *Y*_*J*_ (ascending or descending order depending on the global decision *H*_1_ or *H*_0_, resp., based on weights of the SUs in the previous slot) as follows:

(12) $$\begin{array}{c} Y_{J}=\{\begin{array}{cc} Y_{\left(1\right)}<Y_{\left(2\right)}<\cdots <Y_{\left(M-1\right)}, & H_{1}\\ Y_{\left(M-1\right)}<Y_{\left(M-1\right)}<\cdots <Y_{\left(1\right)}, & H_{0}, \end{array} \end{array}$$

where, in case of *H*_1_, *Y*_(1)_ and *Y*_(*M*−1)_ are the min⁡⁡(*Y*_*i*_^*o*^) and max⁡⁡(*Y*_*i*_^*o*^), respectively, whereas, in case of *H*_0_, *Y*_(1)_ and *Y*_(*M*−1)_ are the max⁡⁡(*Y*_*i*_^*o*^) and min⁡⁡(*Y*_*i*_^*o*^), respectively. In addition to minimizing effect of the SUs with either a faulty sensor or a continuous weak channel due to deep fading, the ascending order suppresses the effect of AF and AO types of MUs, whereas the descending order suppresses the effect of AB and AO types of MUs by assigning low reliability to them. The *M* − 1 SUs in set *Y*_*i*_^*o*^ are assigned normalized reliabilities according to the following two equations:

(13) $$\begin{array}{c} r_{il}^{o}=\underset{J\in \left(M-1\right)}{\arg}\left(Y_{\left(J\right)}=y_{l}\;|\;y_{l}\in Y_{i}^{o},l\neq i\right),\\ R_{li}=\{\begin{array}{cc} \frac{r_{il}^{o}\times 2}{M\left(M-1\right)}, & l\neq i\\ 0, & l=i. \end{array} \end{array}$$

*R*_*li*_ is an *M* × *M* matrix where the diagonal elements are zeros showing exclusion of the *i*th SU in the assignment of weights. Each row of the matrix shows the reliability given to *i*th SU when the other SUs are excluded one at a time. Finally, normalized weight of the *i*th SU is computed by adding elements of the *i*th row of the matrix (all weights assigned to the *i*th SU by others except himself, i.e., numerator in (14)) and divided by the summation of all rows (denominator in (14)), as given by the following equation:

(14) $$\begin{array}{c} w_{i}=R_{i}=\frac{\sum_{l\;=\;1,l\;\neq \;i}^{M}R_{il}}{\sum_{n\;=\;1}^{M}\sum_{l\;=\;1,l\;\neq \;n}^{M}R_{nl}}. \end{array}$$

The reliability of a user is stored in the database (reliability queue) at the FC and is also communicated to the user in the encrypted form as its identification tag (IT) for future use along with the global decision:

(15) $$\begin{array}{c} Q\left(i,k\right)=R_{i},\\ \text{I}{\text{T}}_{k}^{i}=R_{i}, \end{array}$$

where *Q*(*i*, *k*) shows the *k*th slot of the *i*th queue and IT_*k*_^*i*^ is the IT assigned to the* i*th user in the current slot. We assume that only legal SUs know the decryption key, which is updated and exchanged periodically between FC and legal SUs, which enables them to successfully decrypt the IT. In the next time slot, each SU transmits its local sensing result along with the previously decrypted reliability (IT). The FC first applies the MU* screening test *by checking the SU's reported IT with the reliability stored in the corresponding slot for the user in its own database *Q*(*i*, *k* − 1). If a mismatch is found, the FC will declare the user as an MU. Further, the current input (sensing result) from that SU is discarded, and no future reports will be accepted from him:

(16) $$\begin{array}{c} \text{S}{\text{U}}_{i}=\text{MU},\;\text{if}\;\text{I}{\text{T}}_{k-1}^{i}\neq Q\left(i,k-1\right), \end{array}$$

where IT_*k*−1_^*i*^ is the IT reported by the *i*th user.

If an MU is smart enough to deceive the FC by clearing the MU screening test, which is possible only if the MU produces exactly the same reliability as is assigned to a legal user in the previous slot, then the FC performs a* reliability test* to detect MUs and consistently unreliable SUs. The reliability test is comparatively slower because data from the past few slots must be gathered in order to identify the behavior and evaluate the credibility of the user. The purpose of the reliability test is to detect consistently unreliable sensors so that their results can be ignored. In going against the global decision, an MU will also be among the most consistent producers of unreliable results and will thus be stopped after a few slots.

The consistently unreliable SUs are identified by finding the cumulative reliability which is computed by adding the previously stored *K* slots reliabilities as

(17) $$\begin{array}{c} R_{i}^{\text{cum}}=\overset{K}{\underset{j\;=\;1}{\sum }}R_{j}, \end{array}$$

where *j*is the index for slots. The SUs with a cumulative reliability smaller than a predetermined reliability threshold, *λ*_*R*_, are discarded:

(18) $$\begin{array}{c} r_{i}=\{\begin{array}{cc} 1, & R_{i}^{\text{cum}}<\lambda_{R}\\ 0, & R_{i}^{\text{cum}}\geq \lambda_{R}, \end{array} \end{array}$$

(19) $$\begin{array}{c} r=\overset{N}{\underset{i}{\sum }}r_{i}, \end{array}$$

where *r* is the number of users that have unacceptable reliabilities that includes both unreliable and malicious users. Finally, only the remaining users, *M* = *N* − *r*, are considered by the fusion center when making a global decision. The final decision is dependent upon the global threshold and weighting coefficients (reliabilities).

#### 3.1.2. Proposed Scheme II

In this scheme, computations are further simplified. Instead of computing the reliability for each user based on previous results, reliability (renewed in every time slot) is randomly assigned to each user by the FC. The random reliability (RR) is used as IT for the SU and is also stored in the database of the FC for future decisions as

(20) $$\begin{array}{c} \text{I}{\text{T}}_{k}^{i}=Q\left(i,k\right)=\text{R}{\text{R}}_{i}, \end{array}$$

where RR_*i*_ is the random reliability assigned to the *i*th SU and is stored at *Q*(*i*, *k*). The global decision and the respective IT values are communicated to the SUs at the end of each time slot.

Since soft fusion rule is used for global decision in this scheme, therefore, all SUs report their current local observations along with the previously assigned IT (in the decrypted form) to the fusion center, where they are combined with equal weights and a global decision is made about the status of the primary signal. If the IT sent by an SU does not match with the recently (previous slot) stored IT in the reliability queue at the FC, that SU is deemed to be malicious. On the other hand, if a match is found, then the unreliability of the SU is computed. If the local observation does not match with the global decision, the reliability of that particular SU is decreased. In other words, the unreliability, *U*_*i*_, of that SU is increased:

(21) $$\begin{array}{c} U_{i}=U_{i}+\left(Z⊕y_{i}\right), \end{array}$$

where *Z* and *Y*_*i*_ are the 1-bit global and local decisions, respectively, and ⊕ is the exclusive-OR operation that produces 1 when local and global decisions are different and produces 0 otherwise. For computation of the unreliability, 1-bit global and local decisions are considered by the FC, whereas soft fusion rule is used for the global decision. The 1-bit local decision of each user is computed by the fusion center based on the reported observation of the respective user. We assume the same threshold for all SUs to get the 1-bit local decision at the FC.

If the MU screening test fails (i.e., MU produces exactly the same IT as that stored in the queue), then the MU is detected by the reliability test because MU produces a result that frequently deviates from the actual status of the primary signal (global decision). Every time the MU reports a deviant result, its unreliability will increase which occurs more frequently than a user in fading or shadowing. An SU (it may be an MU or a normal SU producing consistently wrong results due to the channel condition or sensor malfunctioning) is stopped from sending reports to the FC when its unreliability reaches a predefined threshold. Only the remaining users that are reliable in terms of generating accurate results contribute to determining the PU status. The dropped SU, represented by SU_*D*_, is not involved in future global decisions and is determined by the following equation:

(22) $$\begin{array}{c} \text{S}{\text{U}}_{D}=\arg\underset{i=1,2,\ldots ,M}{\max}\left(U_{i}\right)\;\text{if}\;U_{\text{thr}}\leq \underset{i=1,2,\ldots ,M}{\max}\left(U_{i}\right). \end{array}$$

In this scheme, the decision of dropping an unreliable SU and MU is taken by the FC.

#### 3.1.3. Proposed Scheme III

The unreliability in this scheme is computed by every SU individually by comparing the local and global decision. To be consistent with the previous schemes we use soft decision approach in this scheme. However, hard decision rule will be more befitting for this scenario. Three types of users are considered here: honest, dishonest, and malicious. Honest users are those who stop reporting when their unreliability exceeds a certain value. In the case of honest users, with time, only reliable users that are less than the total number of users contribute to the detection of the primary signal. Dishonest users continue reporting their untrusted observations even if their unreliability exceeds the threshold. Users with malicious behavior continuously send false data irrespective of the real status of the primary signal and thus severely degrade the detection performance of the network. Dishonest and MUs try to falsify results so as to suit their own selfish interests. As the decision of disengagement from the network is taken at the user level, this approach has no solution for dealing with MUs. Only consistently unreliable users (those with malfunctioning sensor or in deep fades) are restricted. The FC relies on the honesty of SUs and accepts reports from all users. In an environment composed entirely of honest users, consistently unreliable users disengage themselves from reporting when their unreliability reaches a certain limit.

In this approach, each SU performs local sensing, sends its observation to the FC, and waits for the global decision. If the received global decision is different from the local decision, the SU increments its unreliability according to (18). An SU remains in the network as long as its unreliability does not exceed a certain threshold similar to (19). In contrast to schemes I and II, no IT or other reliability calculations are used in scheme III, which makes the approach simple and fast. At any given time, there will be *M* reliable nodes in the CR network. In the case of all honest users, *M* is normally smaller than *N* because unreliable SUs leave the network, thereby keeping the calculations simple and the CR network manageable. If both malicious and honest users are present, the number of users will be less than *N* but greater than *M*. In the case of all dishonest users, the number of users remains fixed and is equal to the total number of users, *N*. Computational simplicity is the main advantage of this strategy; however its disadvantages include lack of control over the MU and unreliable users, as the decision is taken at the SU and results in an increased number of users when they are dishonest.

## 4. Simulation Results 

In this section, we use simulations to compare our proposed strategies with the Chair-Varshney and conventional cooperative spectrum sensing schemes; schemes I and II are considered. In scheme III, the effect of dishonest and MUs is compared with that of honest users. The effects of always opposite MU, always busy MU, always free MU, and *α*MU with *αH*_1_ and (1 − *α*)*H*_0_ are illustrated in the simulations. We evaluate the detection performance of our proposed schemes by plotting receiver operating characteristics (ROC) curve. The simulation parameters are summarized in Table 1.

### 4.1. Results of Proposed Scheme I

**Figure 3:**
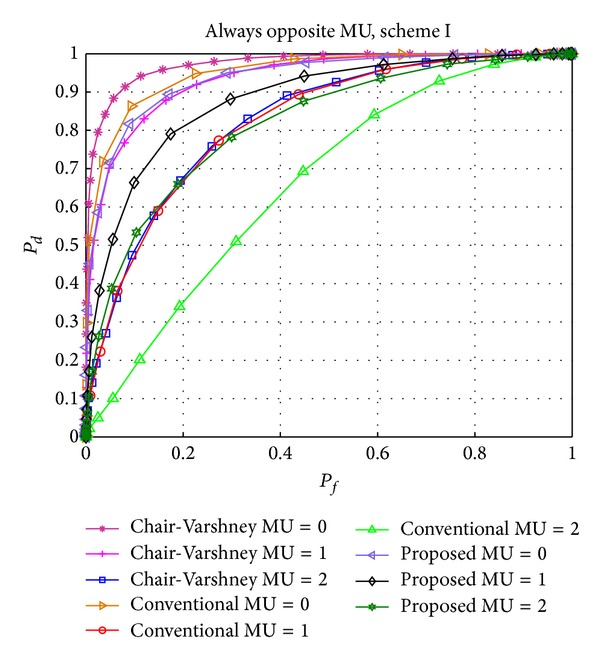
Performance comparison of the Chair-Varshney, conventional CSS, and proposed scheme I with always opposite (AO) MUs.

Figure 3 shows the detection performance of our proposed scheme I, Chair-Varshney (CV) rule, and conventional CSS scheme under the effect of zero, one, and two MUs of the AO type. It is clear from the figure that as the number of AO MUs increases, detection performance of all schemes decreases. By observing the figure it is evident that the detection performance of the Chair-Varshney rule drops quickly when the number of MUs increases to two. Chair-Varshney is the optimum rule but the detection performance of our proposed scheme matches the CV rule for two MUs. Conventional CSS is most severely affected by the AO MUs. When there is no MU, our proposed and conventional CSS schemes both show almost similar results, but our proposed scheme has the advantage of utilizing a smaller number of users. By introducing malicious users (i.e., one or two MUs), our proposed scheme exhibits more robustness and efficiency compared to the conventional CSS. It is also evident from the figure that Chair-Varshney is the optimal detection scheme and provides an upper bound for the other schemes when there is no MU. However, it has a disadvantage in that all users, including consistently unreliable and malicious users, are considered. Our proposed scheme has the advantage of using fewer users for the global decision which is shown in Figure 9.

**Figure 4:**
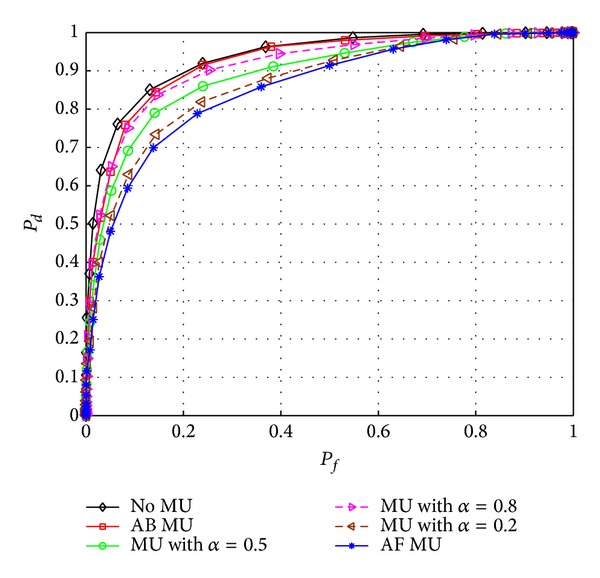
Effect of the AB, AF, and MU that transmits high signal with probability *α* and low signal with probability 1 − *α* on proposed scheme I.

Figure 4 shows the detection performance of the proposed scheme I when there is one AB, AF, or *α*MU. *α*MU is an MU that transmits high signal (*H*_1_), that is, behaving like AB, with probability *α*, and low signal (*H*_0_), that is, behaving like AF, with probability 1 − *α*. To differentiate the effect of AB and AF, *P*(*H*_1_) is set to 0.7 and *P*(*H*_0_) is set to 0.3 such that AF could produce more deviating results compared to AB MU. The detection performance curve of *α*MU is sandwiched between that of AF and AB MU types for 0 < *α* < 1. On one extreme, such MU behaves like AF when *α* = 0 and on the other extreme it behaves like AB when *α* = 1. The effect of such MU is shown in Figure 4 for *α* = 0.5, 0.8, and 0.2, respectively. The performance curve of *α*MU lies in the middle of AF and AB MUs for *α* = 0.5. For *α* = 0.8, the curve of *α*MU is shifted toward AB and for *α* = 0.2 it is shifted towards AF MU.

### 4.2. Results of Proposed Scheme II

This scheme uses a different approach to identify malicious and unreliable users. The advantage of this scheme is its computational simplicity. With a simple approach and without computing reliability for each user, it shows almost similar results with the previous approach. However, the disadvantage is that more users are considered for global decision in this scheme shown by Figure 9.

**Figure 5:**
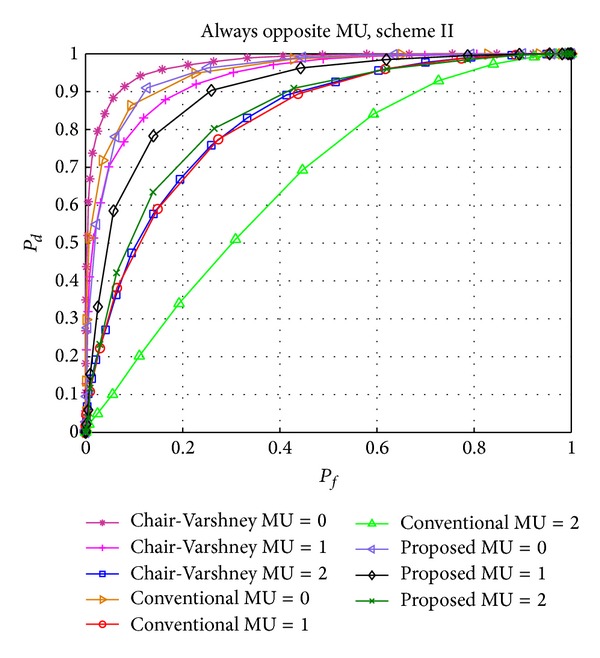
Performance comparison of the Chair-Varshney, conventional CSS, and proposed scheme II with always opposite (AO) MUs.

Figure 5 shows the effect of AO MUs on the performance of the examined schemes. Similar to Figure 3, conventional CSS exhibits the worst performance, in terms of detection performance and the number of users considered for global decision, when exposed to AO MUs. The performance of our proposed method improves to that of the Chair-Varshney approach when the number of MUs increases to two, even though our scheme has the advantage of requiring fewer users (shown in Figure 9).

**Figure 6:**
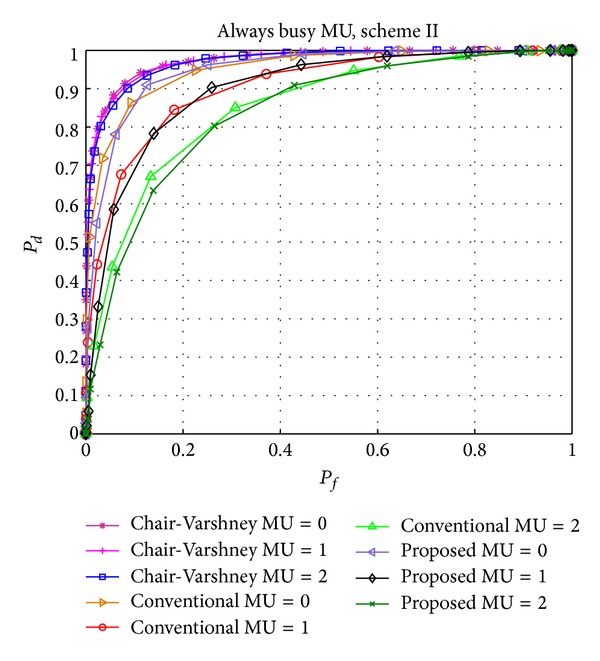
Performance comparison of the Chair-Varshney, conventional CSS, and proposed scheme II with always busy (AB) MUs.

**Figure 7:**
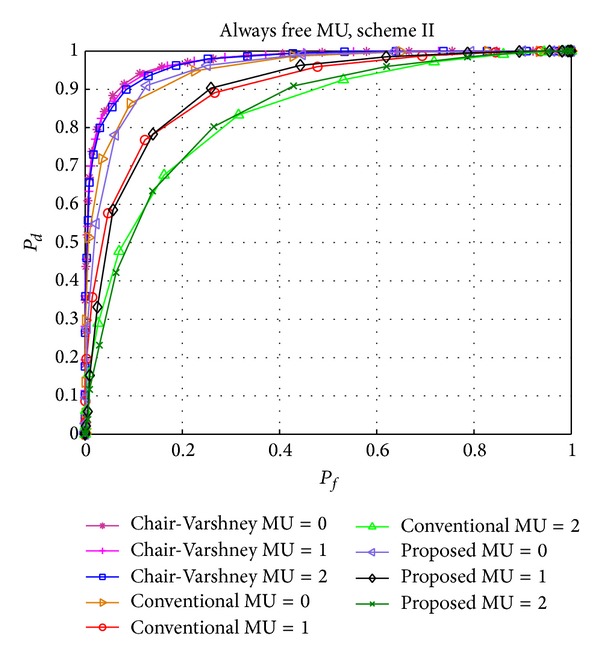
Performance comparison of the Chair-Varshney, conventional CSS, and proposed scheme II with always free (AF) MUs.

Figures 6 and 7 show the effect of AB and AF type of MUs, respectively, on the performance of the Chair-Varshney approach, conventional CSS, and our proposed scheme II. The detection performances of the latter two methods are almost similar due to the following reasons. First, the number of MUs considered is very few compared to legal and reliable users. The detection performance of conventional CSS will be severely affected if the number of MUs is increased. Secondly, due to the equal probabilities of *H*_1_ and *H*_0_, AF and AB MUs show the same effect on the detection performance. Lastly, if the probability of PU arrival is high and AB MUs are present in the network or the idle probability of the PU is high and AF MUs are present in the network, then the effect of AB and AF type of MUs will be low because most of the time the actual status of the PU and sensing report of the MU will be similar which has comparatively less effect on the detection performance. The advantage of our scheme includes the fewer number of (reliable) users that are taken into account for a global decision, as demonstrated in Figure 9 where the average number of users is equal to the total number of users in the case of conventional CSS but fewer for our proposed scheme. This number continues to decrease as the number of MUs increases.

### 4.3. Results of Proposed Scheme III

As discussed in Section 3.1.3, detection performance with this strategy depends on honesty of the users. In the case of dishonest users, every user attempts to influence the global result by showing himself to be reliable (in fact false reliable). All users, including honest users, report their sensing observation to the FC. If the number of dishonest users is small compared to the number of honest users, then the effect of the former will be minimal.

**Figure 8:**
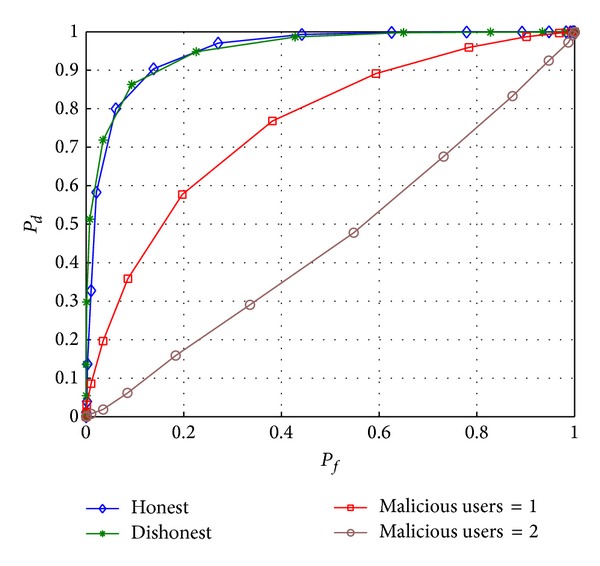
Performance comparison of honest, dishonest, and malicious users in scheme III.

Figure 8 shows the performance comparison for the case that honest users exist only, the case that dishonest users are mixed with honest users, and the case that MUs are mixed with honest users. It is clear from the figure that similar sensing performance is achieved in the honest and dishonest cases because there are very few dishonest users present. In the case that all users are honest, the average number of users will always be less than the number of users when all of them are dishonest. No control over MUs is achieved in this scheme and thus, MUs severely contaminate the sensing performance. As is clearly evident from the figure, by increasing the number of MUs from 1 to 2, a high deterioration is observed in the detection performance.

### 4.4. Comparison of the Average Number of Users for Global Decision by Our Proposed Schemes and Other Schemes

**Figure 9:**
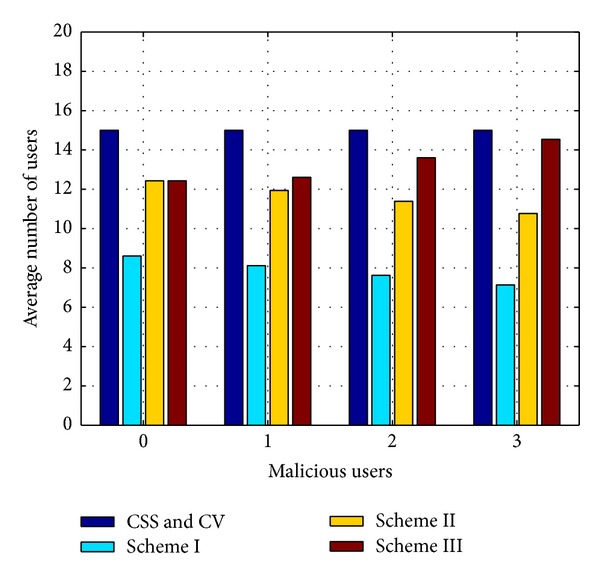
Average number of users for global decision in conventional CSS, CV (Chair-Varshney), and our proposed schemes for numbers 0, 1, 2, and 3 of malicious users.

Figure 9 shows the average number of SUs considered for the global decision when conventional CSS, Chair-Varshney rule, and our proposed schemes are considered in the presence of zero, one, two, and three MUs. It is observed from the figure that the average number of users, in the case of the conventional CSS and Chair-Varshney rule, is equal to the total number of SUs in the network. However, fewer users (that even decrease further with increasing MUs) are used for global decision in our proposed schemes. Scheme I outperforms all the other schemes in terms of the number of users and shows almost similar detection performance to scheme II. The average number of users in scheme I decreases as the number of MUs increases because the MUs are successfully blocked by scheme I which reduces the number of users. It is also visible from the figure that the average number of users in scheme II is more than that in scheme I. The reason is that in scheme I each user has a relative weight depending on the accuracy of the result. Thus, unreliable users get less weight and are suppressed from the network which decreases the average number of users. In contrast, all users (reliable and unreliable) have equal weights in scheme II and are excluded only when their unreliability reaches a certain limit. In scheme III, the average number of users in the dishonest case is 15 (total users), while for the honest case it is less than maximum but increases with the number of MUs because MUs pretend to be honest and remain in the network. Since schemes II and III use unreliability to ignore a user, therefore the number of users, when there is no MU, is equal in both schemes. Scheme I and scheme II use 43% and 17% reduced users, respectively, to show similar detection performance to that of conventional CSS when there is no MU. The detection performance improves further with the improvement of users (decrease in the number of users) of 52% and 28% in scheme I and scheme II, respectively, when there are two MUs in the network. However, the number of users considered for global decision in scheme III is 17% and 3% less than the conventional and CV schemes when MU = 0 and MU = 2, respectively.

## 5. Conclusion

In this paper, we have proposed simple but effective schemes to combat MUs and control consistently unreliable users. Nonuniform reliability and reliability-based IT are used to isolate unreliable and malicious users in scheme I. Unreliability and randomly chosen IT are used to control unreliable and MUs in scheme II. In scheme III, honest users stop sending reports when their trust level decreases below a certain threshold. The results produced by consistently unreliable users due to either permanent deep fades or sensor malfunctioning are restricted so as to minimize their effect on the global result. Restricting the number of users to only those that are reliable makes the network manageable and reduces the computational cost and other overhead.

We intend to extend this work in the future by analyzing latency and energy consumption of the CR network with our proposed schemes.

## Figures and Tables

**Table 1:** System parameters.

Description	Symbol	Value
Number of iterations	*l*	5000
Number of SUs	*N*	15
PU busy probability	*p*_*H*_1__	0.5
Sensing duration	*T*_*s*_	1 ms
Sampling frequency	*f*_*s*_	300 KHz
Number of samples	*S*	600
Signal-to-noise ratio	*γ*	[−25 dB, −10 dB] with 1 dB decrement
Maximum number of MUs	*L*_max⁡_	3
Minimum number of reliable SUs	*M*_min⁡_	5
Size of queue	*Q*	15 × 50
Depth of each user queue (each row in *Q*)	*K*	50
Unreliability threshold	*U*_thr_	10
Probability of H1 transmission by *α*MU	*α*	[0.2, 0.5, 0.8]
